# Ayurveda Management of Menorrhagia (Raktapradara): Protocol for a Randomized Controlled Trial

**DOI:** 10.2196/60801

**Published:** 2025-03-31

**Authors:** Shivshankar Rajput, Shweta Mata, Upma Saxena, Sarada Ota, Bharti Gupta

**Affiliations:** 1 Central Council for Research in Ayurvedic Sciences Central Ayurveda Research Institute New Delhi India; 2 Department of Obstetrics & Gynecology Vardhman Mahavir Medical College & Safdarjung Hospital New Delhi India; 3 Central Council for Research in Ayurvedic Sciences Central Ayurveda Research Institute Bhubaneshwar India

**Keywords:** Ashokarishta, Trinakantamani pishti, tranexamic acid, menorrhagia, Raktapradara

## Abstract

**Background:**

In India, heavy menstrual bleeding or menorrhagia (*Raktapradara*) constitutes about 15% to 20% of all gynecological admissions in an institution. Of these, 43% of patients are aged 20-40 years. This condition is worsening because of the high prevalence of anemia among Indian women. Menorrhagia can have a significant impact on women’s lives. Medical treatment is usually the first choice in excessive bleeding, but it reduces menstrual blood loss by only 50%, and up to 50% of women undergo surgical treatment within 5 years. However, none of these treatments proved their definite efficacy in spite of the high price and side effects. This condition presents a major financial burden on health care services. In Ayurveda, encouraging work has been done on the compound drug *Ashokarishta*, and the drug *Trinakantamani pishti* is indicated in Ayurvedic classics and the *Ayurvedic Formulary of India*. Also, these medicines have been used in Ayurvedic practice for a long time. However, no clinical trial has been carried out on these formulations.

**Objective:**

The primary objective is to evaluate the efficacy of Ayurvedic intervention in the management of menorrhagia, and the secondary objective is to assess the efficacy of Ayurvedic intervention on the quality of life of the women with menorrhagia.

**Methods:**

This ongoing study is an open-label, interventional, randomized controlled trial, with a sample size of 140 in the treatment and control groups combined (including 20% dropouts), and will be carried out within the duration of 36 months. Participants in the treatment group will receive Ayurvedic treatment, that is, 20 mL of *Ashokarishta*, 250 mg of *Trinakantamani pishti*, and 1 iron and folic acid tablet (100 mg of elemental iron and 1.5 mg of folic acid) twice a day orally for 3 months. Participants in the control group will receive a 500-mg tranexamic acid tablet thrice a day for 7 days from the first day of menses for 3 cycles and 1 iron and folic acid tablet twice a day orally for 3 months. The primary outcomes are changes in the amount of uterine bleeding evaluated by the Pictorial Blood Loss Assessment Chart, changes in the duration of bleeding, and attainment of a normal quantity of blood loss during the interval of cycles. The secondary outcome is changes in the Menorrhagia Impact Questionnaire.

**Results:**

As of December 2024, a total of 79 patients have been enrolled. Data analysis should be completed by February 2026. The study will be reported following standard guidelines for reporting randomized controlled trials. Clinical results will be disseminated through conferences and peer-reviewed publications in a relevant journal.

**Conclusions:**

The Ayurvedic approach may provide an evidence-based therapeutic tactic for the management of menorrhagia.

**Trial Registration:**

Clinical Trial Registry India CTRI/2023/05/052929; https://tinyurl.com/3cd6mxrn

**International Registered Report Identifier (IRRID):**

DERR1-10.2196/60801

## Introduction

Normal menstrual bleeding is cyclical, with 3-5 days in durations and 50-60 mL of bleeding in normal color, as described in Ayurvedic classics. However, when the normal menstrual bleeding pattern is altered in reference to duration, amount, color, and cycle, the conditions are called *Artavadushti*; *Raktapradara* is one of them, and it is comparable to menorrhagia. In India, menorrhagia constitutes about 15% to 20% of all gynecological admissions in an institution. Of these, 43% of patients are aged 20-50 years. This condition is worsening because of the high prevalence of anemia among Indian women [1]. Heavy menstrual bleeding (HMB) can have a significant impact on women’s lives. HMB or menorrhagia is clinically deﬁned as greater than or equal to 80 mL blood loss per menstrual cycle [2,3]. It is, however, the woman’s perception of her own menstrual loss that is the key determinant in a referral and, indeed, subsequent treatment. In allopathic medicine, various medical treatment options are available, but many women proceed to surgery due to treatment failure or hormonal side effects. Surgery introduces the risk of bowel, bladder, and ureteric damage, as well as hemorrhage, infection, and even death [4]. So, there is a clear unmet clinical need for better medical treatments for this benign but incapacitating condition. *Ashokarishta* is a compound drug, consisting of 14 drugs described in the *Stree rogadhikara* (female disorders) section of the *Bhaishajya Ratnavali* book of Ayurveda [5]. The *phalashruti* (benefits at the end of the composition) of this medicine says that, by using the medicine for more than one month, it cures pain due to menorrhagia, fever, several types of hemorrhages, hemorrhoids, etc. *Trinakantamani pishti* [6,7] is a drug also mentioned for *Raktapradara* (HMB or menorrhagia) in Ayurvedic classics. As the effect of this medicine is mainly in bleeding disorders, it was decided to evaluate its efficacy in *Raktapradara* (Table 1). Some encouraging work has been done on the effect of *Ashokarishta* [8-10] on *Raktapradara*, but no work has been carried out on *Trinakantamani pishti* along with *Ashokarishta*. Tranexamic acid is a safe and effective form of medical therapy in women with menorrhagia; it also increases the quality of life in these women [11]. So, this study is planned to evaluate the efficacy of Ayurvedic treatment modalities in the management of menorrhagia and to assess the efficacy of Ayurvedic intervention on the quality of life of the women with menorrhagia, compared to the control intervention (tranexamic acid).

**Table 1:** Details of the investigational products.

Drug and components		Botanical name	Part used	Quantity
***Trinakantamani pishti***				
	1. Trinakantamani	—^a^	—	1 part
	2. Distilled rose water	—	—	As needed for *mardana* (triturate)
**Ashokarishta**				
	1. Ashok	*Saraca asoca* L.	Stem bark	100 parts
	2. Water for decoction (*kashaya*), reduced	—	—	256 parts
	3. Guda (jaggery)	*Saccharum officinarum*	—	200 parts
	4. *Dhataki*	*Woodfordia fruticosa* (L.) Kurz.	Flower	16 parts
	5. *Ajaji*	*Nigella sativa* L.	Fruit	1 part
	6. *Mustak*a (*musta*)	*Cyperus rotundus* Linn.	Rhizome	1 part
	7. *Sunthi* (ginger)	*Zingiber officinale* Roxb.	Rhizome	1 part
	8. *Darvi* (*daruharidra*)	*Berberis aristata* DC.	Stem	1 part
	9. Utpala	*Nymphaea stellata* Willd.	Flower	1 part
	10. *Haritaki*	*Terminalia chebula* Retz.	Whole plant	1 part
	11. *Bibhitaka*	*Terminalia bellirica* (Gaertn.) Roxb.	Whole plant	1 part
	12. *Amalaki*	*Phyllanthus emblica* L.	Whole plant	1 part
	13. *Amrasthi* (*Amra*)	*Mangifera indica* L.	Endosperm	1 part
	14. *Jiraka* (*sveta jiraka*)	*Cuminum cyminum* L.	Fruit	1 part
	15. *Vasa*	*Adhatoda vasica* Nees.	Root	1 part
	16. *Chandana* (*sveta candana*)	*Santalum album* Linn.	Heart wood	1 part

^a^Not applicable.

## Methods

### Study Setting

This study will be conducted at Vardhman Mahavir Medical College and Safdarjung Hospital, New Delhi, India.

### Eligibility Criteria

#### Inclusion Criteria

Participants will include those aged 20-50 years with menorrhagia—that is, regular (21-35 days) cycle with HMB loss, subjectively or objectively defined (Pictorial Blood Assessment Chart [PBAC] score of more than 100 points) [12], or prolonged bleeding (bleeding more than >7 days) for 3 consecutive cycles—who are willing and able to participate in the study.

#### Exclusion Criteria

Participants with any of the following will be excluded: anemia (hemoglobin level <7%); hypertension; diabetes mellitus; hepatic or renal disease; cardiac disease; organic lesions of the reproductive tract due to conditions such as tuberculosis, carcinoma, and congenital deformities; pelvic inflammatory disease or cervicitis; evidence of malignancy, cervical intraepithelial neoplasia, or cervical carcinoma; history of untreated sexually transmitted disease or being HIV positive; past history of atrial fibrillation, acute coronary syndrome, myocardial infarction, stroke, or severe arrhythmia in the last 6 months; lactating women; current or previous use of oral contraceptive pills, glucocorticoids, antiandrogens, ovulation induction agents, antidiabetic drugs, antiobesity drugs, or other hormonal drugs recently (unless there is a washout period of 1 month); hypersensitivity to the trial drugs or any of their ingredients; currently participating in any other clinical trial or participated in past 6 months; or any other condition that the principal investigator thinks may jeopardize the study.

### Study Interventions (Investigational Products)

Participants in the treatment group will receive 250 mg of *Trinakantamani* *pishti* twice a day orally before meals, 20 mL of *Ashokarishta* twice a day orally before meals, and 1 iron and folic acid tablet (100 mg of elemental iron and 1.5 mg of folic acid) twice a day orally after meals for 3 months. The treatment group’s medicines (investigational products) will be procured from Good Manufacturing Practices–certified pharmacies, namely, Indian Medicines Pharmaceutical Corporation Ltd. and Central Ayurveda Research Institute, along with the Certificate of Analysis to ensure the quality and safety of the medicines. The study drugs will be kept in a secure place and will only be supplied to the participants under the guidance of the investigator. A record will be maintained for the drug dispensed. Any discrepancies between amounts dispensed and returned will be explained.

Participants in the control group will receive a 500-mg tranexamic acid tablet thrice a day for 7 days from the first day of menses for 3 cycles and 1 iron and folic acid tablet twice a day orally for 3 months.

### Strategies to Improve Adherence to Study Protocol

The compliance of taking trial drugs will be assessed at each visit during the follow-ups (30-day intervals) by assessing the approximate quantity consumed. The participants will be asked to return the empty container of medicine at the time of each follow-up visit. Repeated reminders will be given over the phone or through family members and project staff for regular taking of medicine.

### Outcome Measures

#### Primary Outcome Measures

Primary outcome measures are changes in the amount of uterine bleeding evaluated by the PBAC, changes in the duration of bleeding, and the attainment of a normal amount of blood loss during the interval of cycles, evaluated at baseline, the end of every cycle up to 3 cycles, and follow-up visits.

#### Secondary Outcome Measure

The secondary outcome measure is changes in the Menorrhagia Impact Questionnaire (MIQ) [13], evaluated at baseline, the end of intervention period, and follow-up visits.

#### Safety Outcomes

The safety of the trial intervention will be evaluated by recording the incidence of adverse events on every scheduled follow-up visit. All adverse events during the study timeline will be recorded and monitored per the Good Clinical Practice Guidelines. The investigator will report the same to the institutional ethics committee and the sponsors at the earliest opportunity. To assess the safety, hematological and biochemical investigations such as complete blood count and liver and kidney function tests will be done at baseline and the end of the intervention period.

### Sample Size

To calculate the sample size, we anticipated a difference of 70 units in average PBAC score after treatment between the two groups (*Trinakantamani* *pishti* along with *Ashokarishta* vs tranexamic acid tablet), with an SD of 137 units based on the results of the previous study [14]. With 95% confidence level (α=.05) and 80% power, the number of patients to be enrolled in the study was calculated using the following formula:

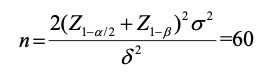



where *Z*_1 – α/2_=1.96, *Z*_1 – β_=0.84, σ=137, and δ=70.

Expecting a dropout rate of 15%, the number of patients is estimated to be 60 + 9 = 69. Thus, approximately 70 patients should be enrolled in each group.

### Randomization and Allocation

A randomization chart will be generated with the help of computer-generated random numbers. Participants will be randomized in a ratio of 1:1 to either of the two groups. The randomization schedule will be strictly controlled and remain with the biostatistician/data analyst. Sequentially numbered, sealed, opaque envelopes will be used to conceal the allocation.

### Study Procedure

The participants will be screened for menorrhagia as per the inclusion criteria. Prior to any trial-related activity, the investigator will give the verbal information and written information in a printed form about the trial to the participant, parents, or guardians to read and understand. The investigator would ensure that the participants are fully informed about the aims, procedures, discomforts, and expected benefits of the trial. It must be emphasized that participation is voluntary, and participants have the right to discontinue the trial at any time without any prejudice. A voluntary, signed informed consent will be obtained from the participants prior to any clinical trial–related procedure.

**Figure 1:**
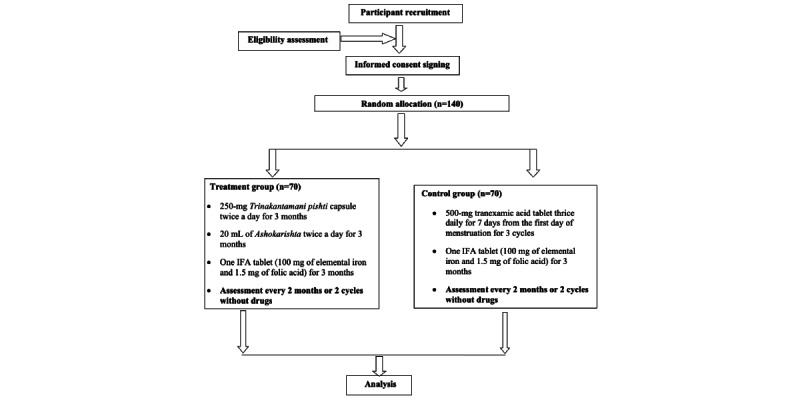
Flowchart of the study procedure.

After the screening, if the participants are found to be suitable, then they shall be enrolled in the study after signing the consent form. The required investigations will be carried out, and subsequently, within one week or as soon as laboratory values are received and found to be within permissible limits, the participants will be enrolled in the study, and that day will be considered visit 1 or the baseline visit. The treatment will be given to the participants after fulfilling all the formalities as per the protocol. The subsequent follow-ups will be on the 30th day (visit 2), the 60th day (visit 3), the 90th day (visit 4), the 120th day (visit 5), and the 150th day (visit 6; ie, completion of active treatment). Participants will visit the study site 6 times during the trial. The details of the activity during each visit are presented in Table 2 and Figure 1.

**Table 2:** Study schedule.

Components	Screening	Baseline (visit 1)	30th day (visit 2)	60th day (visit 3)	90th day (visit 4)	120th day (visit 5)	150th day (visit 6)
Informed consent	✓						
Demographics and medical history		✓					
Laboratory investigations	✓		✓		✓		
Assessment of clinical signs and symptoms		✓	✓	✓	✓	✓	✓
Assessment of the MIQ^a^		✓	✓	✓	✓	✓	✓
Clinical examination	✓						
Assessment of the PBAC^b^		✓	✓	✓	✓	✓	✓
Assessment of ADRs^c^			✓	✓	✓		
Assessment of drug compliance			✓	✓	✓		
Issue of trial drugs every 20 or 30 days							

^a^MIQ: Menorrhagia Impact Questionnaire.

^b^PBAC: Pictorial Blood Loss Assessment Chart.

^c^ADR: adverse drug reaction.

### Timelines With Deliverables

The total study period spans 36 months and is divided into four distinct phases. During the initial phase, which lasts from months 1 to 6, efforts will focus on procuring standard trial drugs and finalizing laboratory arrangements. Additionally, tasks such as staff recruitment, equipment procurement, and finalization of case report forms (CRFs) will be undertaken. The second phase, covering months 7 to 23, will be dedicated to the recruitment of participants for the study. Subsequently, months 24 to 34 will constitute the treatment period, during which participants will undergo interventions as per the study protocol, with follow-up assessments conducted accordingly. Finally, in the last phase spanning months 35 to 36, data compilation, analysis, report preparation, and publication will be the primary activities, culminating in the dissemination of study findings.

### Laboratory Tests

The laboratory tests (hematological tests, biochemical tests, and ultrasonography) will be conducted in a National Accreditation Board for Testing and Calibration Laboratories–accredited laboratory. Hematology (hemogram, lipid profile, fasting blood sugar and postprandial blood sugar levels, and hemoglobin A_1c_ level); liver function test; kidney function test; venereal disease research laboratory test; HIV test; hepatitis B surface antigen test; ultrasonography (transabdominal sonography or transvaginal sonography); coagulation profile; hormonal test like thyroid profile, serum prolactin test, serum follicle-stimulating hormone test, and serum luteinizing hormone test; routine urine culture; and microscopic urinalysis will be carried out at baseline and the end of the treatment (90th day). Coagulation profile will also be done at the middle of treatment (30th day).

### Data Collection, Management, and Analysis

The data collection will include all information in the CRFs. All participants will be assigned an enrollment number, which will be used on CRFs and electronic databases. Consent forms and CRFs will be stored in locked cupboards, and electronic databases will be password protected. The data will be entered using the double-entry method for accuracy. All data will be accessible to the principal investigator and coinvestigators during and after the study has been completed and will be available to sponsors and monitors as required. Data will be stored for 5 years before being destroyed. In case of dropout, 10% data will be inserted through the imputation technique. Proper documentation will be done to ensure its accurate interpretation and verification. The analysis will be done by the Central Council for Research in Ayurvedic Sciences statistical unit.

### Statistical Methods

Continuous variables will be presented as mean, SDs, medians, and ranges. Categorical variables will be summarized with frequencies and percentages. When inferential analyses will be conducted for continuous variables, they will be tested for normality. Nonparametric methods will be used to compare nonnormal data. Within-group comparison will be done using a paired *t* test (1- or 2-tailed, as suitable), while between-group comparisons will be done using an independent sample *t* test (1- or 2-tailed, as suitable) for the data following normal distribution. Nonnormal data within the group will be compared using the Wilcoxon sign rank test, while between groups comparison will be done by using the Mann-Whitney *U* test. Categorical data between groups will be compared using the chi-square or Fisher exact test. A 2-sided *P* value of <.05 will be considered statistically significant. Per-protocol and intention-to-treat analyses will be carried out at the end of the study. All data will be analyzed using Stata (version 16.1; Stata Corp).

### Ethical Considerations

This research protocol had been reviewed and approved by the institutional ethics committee of Vardhman Mahavir Medical College and Safdarjung Hospital (S. No. IEC/VMCC/SJH/NOTE/2023-Feb/06). Participants will receive an information sheet with the research details given in two languages (Hindi and English). Voluntary signed informed consent will be obtained from the participants before any clinical trial–related procedure. The participants will be informed by the investigator that all trial data recorded will be treated with strict confidence. During documentation and analysis of the trial, the individual participant will only be identified by their enrollment number.

Participant confidentiality will be guaranteed, and only researchers will have access to the data. Participants are nominally compensated for their loss of wages and transportation costs by paying an amount of IND ₹100 (US $1.19) for every visit to the hospital during the study period.

### Data Safety and Monitoring

The safety of the trial intervention will be evaluated by recording the incidence of adverse events on every scheduled follow-up visit. All adverse events during the study timeline will be recorded and monitored per the International Council for Harmonization of Technical Requirements for Pharmaceuticals for Human Use’s Guideline for Good Clinical Practice. The investigator will report the same to the institutional ethics committee and the sponsors as soon as possible.

For facilitating appropriate reference standards for scientific, ethical, and safety issues before the trial begins, this protocol has been developed according to the 2013 SPIRIT (Standard Protocol Items: Recommendations for Interventional Trials) and CONSORT (Consolidated Standards of Reporting Trials) statements [15].

After completion of the study on 25% of the total participants, the data will be analyzed for the safety of the trial drugs. The project monitoring committee will monitor the trial’s progress through review meetings and site visits as per the requirement to ensure strict adherence to the trial protocol and correct completion of the CRF.

### Drug Compliance

The compliance of taking trial drugs will be assessed at each visit during the follow-ups (30-day intervals) by assessing the approximate quantity consumed. The participants with equal to or more than 80% compliance will continue in the study. The participants will be asked to return the empty container of medicine at the time of each follow-up visit. Repeated reminders will be given over the phone or through family members and project staff for the regular taking of medicine.

### Prior and Concomitant Medication

Participants will receive clear instructions not to take any medication other than the trial drugs for any condition without prior consultation with the investigators. The investigator will advise participants to promptly report any additional signs, symptoms, or unusual occurrences and to seek guidance accordingly. Additionally, the investigator will diligently document any other medications taken by the participants for comprehensive recordkeeping.

### Rescue Medication

Permission will be given for the use of rescue medication for alleviating any emergency condition as per the discretion of the investigator. It will be documented in detail in the CRF.

## Results

As of December 2024, a total of 79 patients have been enrolled. Data analysis should be completed by February 2026. The study will be reported following standard guidelines for reporting randomized controlled trials. Clinical results will be disseminated to the public through conference presentations and peer-reviewed publications in a relevant, open-access journal.

## Discussion

### Conclusion

Ayurveda scholars correlate *Raktapradara* with menorrhagia (National Ayurveda Morbidity Code: EL-4) based on the similarities in causes, clinical features, and treatment. This paper describes the protocol for the Ayurvedic management of menorrhagia. We undertook a randomized controlled trial to objectively assess the efficacy of Ayurvedic treatments for the management of menorrhagia and compared it with standard conventional treatment. The changes are assessed objectively by using the PBAC score and MIQ as the outcomes for participants with menorrhagia.

Studies have demonstrated promising results of some Ayurvedic interventions for managing *Raktapradara*. With this study, we aim to observe the effectiveness and safety of Ayurvedic management using the formulations of *Ashokarishta* and *Trinakantamani pishti* in reducing symptoms associated with menorrhagia, such as HMB. If beneficial, this study could contribute significantly to integrative approaches for managing menorrhagia.

### Strengths and Limitations

The strengths of this study include its randomized design and the inclusion of enough participants to allow for adequate statistical power for subgroup analyses. This study uses the PBAC score and MIQ, which are well-validated instruments to assess menorrhagia.

One potential limitation of this study is that the investigators were aware of the participants’ group assignment, and therefore, bias in favor of any particular group cannot be excluded. However, every effort was made to ensure blinding of the participants during the group allocation. An independent evaluator blind to study conditions was responsible for administering all study measures. The protocol may also serve as a reference for the planning of similar clinical trials.

## Abbreviations

CONSORT: Consolidated Standards of Reporting Trials
CRF: case report form
HMB: heavy menstrual bleeding
MIQ: Menorrhagia Impact Questionnaire
PBAC: Pictorial Blood Loss Assessment Chart
SPIRIT: Standard Protocol Items: Recommendations for Interventional Trials

## Data Availability

The findings will be disseminated to the public through conference presentations and published in open-access, peer-reviewed journals. Analyzed data will be made available for interested researchers with a reasonable request after the publication of the results.
